# Creative Engagement and Cognitive Function: Implications for Gerontology Practice

**DOI:** 10.1093/geroni/igaf122.1678

**Published:** 2025-12-31

**Authors:** Janelle Fassi

**Affiliations:** University of Massachusetts Boston, Boston, Massachusetts, United States

## Abstract

This presentation will discuss scholarly work on 1) the relationship between engagement in creative leisure activities and cognitive functioning among older adults and 2) whether education plays a moderating role in this relationship. Significantly, individuals who demonstrated the highest level of creative engagement (four activities) had the best cognitive functioning. In sum, this research highlights the importance of remaining engaged in creative pursuits in later life and is just one example of the innovative ways to bridge gerontology research and practice. The sample (n = 2,462) consisted of adults aged 65 years and older who completed either the 2016 or 2018 waves of the Leave Behind Questionnaire (LBQ) and the 2015 wave of the Consumption and Activities Mail Survey (CAMS) as part of the Health and Retirement Study. Cognitive function was measured using the Telephone Interview for Cognitive Status (TICS-M) and a new conceptualization of creative engagement was created using four leisure activities from the LBQ and CAMS. Results from linear regression models revealed that engagement in creative activities was associated with better cognitive functioning scores. Education (measured in years) was also positively associated with cognitive function; however, education did not have a moderating effect in the relationship between creative engagement and cognitive function.

